# Bovine Tuberculosis in Cattle in the Highlands of Cameroon: Seroprevalence Estimates and Rates of Tuberculin Skin Test Reactors at Modified Cut-Offs

**DOI:** 10.1155/2012/798502

**Published:** 2012-04-01

**Authors:** J. Awah-Ndukum, A. C. Kudi, G. S. Bah, G. Bradley, S. F. Tebug, P. L. Dickmu, H. N. Njakoi, W. N. Agharih

**Affiliations:** ^1^School of Veterinary Medicine and Sciences, University of Ngaoundere, Ngaoundere, Adamawa Region, Cameroon; ^2^Department of Animal Sciences, University of Dschang, Dschang, West Region, Cameroon; ^3^School of Biomedical and Biological Sciences, University of Plymouth, Plymouth, PL4 8AA, UK; ^4^Department of Veterinary Medicine, Ahmadu Bello University, Zaria, Kaduna State, Nigeria; ^5^Institute of Agricultural research for Development (IRAD), Wakwa- Ngaoundere, Adamawa Region, Cameroon; ^6^Institute of Animal Breeding and Husbandry, University of Kiel, Kiel, Germany; ^7^Department of Mathematics and Computer Science, University of Dschang, Dschang, West Region, Cameroon; ^8^Heifer Project International, P.O. Box 467, Bamenda, North West Region, Cameroon; ^9^Delegation of Livestock, Fisheries and Animal Husbandry, North West Region, Cameroon

## Abstract

The aim of this study was to obtain epidemiological estimates of bovine tuberculosis (TB) prevalence in cattle in the highlands of Cameroon using two population-based tuberculin skin test (TST) surveys in the years 2009 and 2010. However, prior to the TST survey in 2010, blood was collected from already chosen cattle for serological assay. Anti-bovine TB antibodies was detected in 37.17% of tested animals and bovine TB prevalence estimates were 3.59%–7.48%, 8.92%–13.25%, 11.77%–17.26% and 13.14%–18.35% for comparative TST at ≥4 mm, ≥3 mm and ≥2 mm cut-off points and single TST, respectively. The agreement between TST and lateral flow was generally higher in TST positive than in TST negative subjects. The *K* coefficients were 0.119, 0.234, 0.251 and 0.254 for comparative TST at ≥4 mm, ≥3 mm and ≥2 mm cut-off points and the single TST groups, respectively. Chi square statistics revealed that strong (*P* < 0.05; *χ* ^2^ > 48) associations existed between seroprevalence rates and TST reactors. The study suggested that using lateral flow assay and TST at severe interpretations could improve the perception of bovine TB in Cameroon. The importance of defining TST at modified cut-offs and disease status by post-mortem detection and mycobacterial culture of TB lesions in local environments cannot be overemphasised.

## 1. Introduction

Bovine tuberculosis (TB) is a zoonotic disease with severe public health significance but it is neglected in Cameroon. The tuberculin skin tests (TSTs) are currently the best available techniques for international field diagnosis of bovine TB in live animals [1, 2] and it is based on delayed hypersensitivity reactions [3]. The single intradermal comparative cervical tuberculin (SICCT) test involving the intradermal injection of bovine tuberculin (BT) and avian tuberculin (AT) at separate sites in the skin of the neck gives more specific results than the single intradermal tuberculin (SIT) test which uses only BT [4, 5]. TST can effectively detect early stages of *M. bovis* infection in cattle and allows for rapid removal of infected animals, limited transmission, and fast eradication of bovine TB [6]. There are OIE-recommended cutoff points of the increase in skin thickness for SICCT-BT and SIT-BT to be positive [3]. However, the OIE-recommended cutoff values were established mainly in developed countries for *Bos taurus *cattle, and different cutoff values are applied according to a particular country's disease status and objective of its disease control programme [4, 7–9]. 

The performance of TST could be affected by environmental factors, host factors (status of immunity, genetics), and nature of the tuberculin used [1, 4, 5, 9]. A perfect cutoff point in a specific geographic area may not be so useful in another environment [1, 4]. Also, the ability of the test to predict positive disease status depends on its sensitivity and specificity and prevalence of the disease in tested population [1]. Anergic animals, animals exhibiting reactions to both avian and mammalian tuberculins, animals in advanced stage of disease, periparturient cows, and animals with confined infection notably in the udder and with localised infection often in the lymphatic glands that has become inactive (latent) have been reported to be poor responders to TST [10]. However, severe interpretations are done in regions or herds where *M. bovis* infection has been confirmed, and SIT-BT reactors may also be subjected to an SICCT-BT test, based on the discretion of the veterinarian [4]. Veterinarians continue to play pivotal roles in inspections of animal (antemortem and postmortem) and animal products, diagnosis of *M. bovis *infected cattle, and impacting of cattle producers in bovine TB eradication programs [11]. Postmortem detection of TB lesions and other bovine TB diagnostic techniques (e.g., gamma-Interferon, ESAT-6 tests, serologic and fluorescence polarization assays) have been used to determine the ability of TST in the diagnosis of bovine TB in cattle in different environmental conditions around the world, including parts of Africa [1, 2, 6, 7, 9, 12–16]. However, TST-negative animals at slaughter with evidence of encapsulated lesions confirmed as caused by *M. bovis *have also been reported [10]. 

TST may demand physical exertion in the field but it is also simple and relatively inexpensive and offers reliable means of screening cattle populations in an entire region [4, 6]. Ancillary tests are being used and/or currently being validated to improve diagnosis and reduce the number of false positive results following TST [1, 2, 6, 7]. Also, rapid and simple immune-chromatographic assays for the serodiagnosis of bovine TB have been developed [17, 18] and proposed as additional tests to the TST for *antemortem* diagnosis [2, 19, 20]. These chromatographic immunoassays employ unique cocktails of selected *M. bovis* antigens as both qualitative captures and detectors of specific antibodies against *M. bovis *in plasma, serum, and whole blood [17, 21]. MPB83, ESAT-6, 14-kDa protein, CFP-10, MPB70, MPT63, MPT51, MPT32, MPB59, MPB64, Acr1, PstS-1, *M. bovis *purified protein derivatives, ESAT-6/CFP10 fusion protein, 16-kDa alpha-crystallin/MPB83 fusion protein, and *M. bovis *culture filtrate have been identified as the common seroreactive antigens in bovine TB [17, 18, 22]. The bound antibodies are visualized with the naked eye as colour band at the test device within some minutes of application [17, 21]. The assay requires no specific expertise or equipment, and the test kit may be kept without the need for refrigeration [17, 18, 21].

There are scanty reports of bovine TB prevalence in Cameroon, modifications of the OIE standards of TST applied elsewhere have been used to estimate the disease status in cattle in the country, and the findings have varied widely, even for the same sites [23–27]. This study was carried out to investigate bovine TB prevalence in cattle in the highlands of Cameroon through seroprevalence estimations, rates of TST reactors at modified cutoff points, and the epidemiological usefulness of the proposed screening algorithms. TST data of tested cattle in the years 2009 (*n* = 2, 853) and 2010 (*n* = 1, 381) were reanalyzed, and the epidemiological implication for applying TST at various cutoff points for a predominantly Zebu cattle population was discussed. 

## 2. Materials and Methods

### 2.1. Study Area and Population

**Figure 1:**
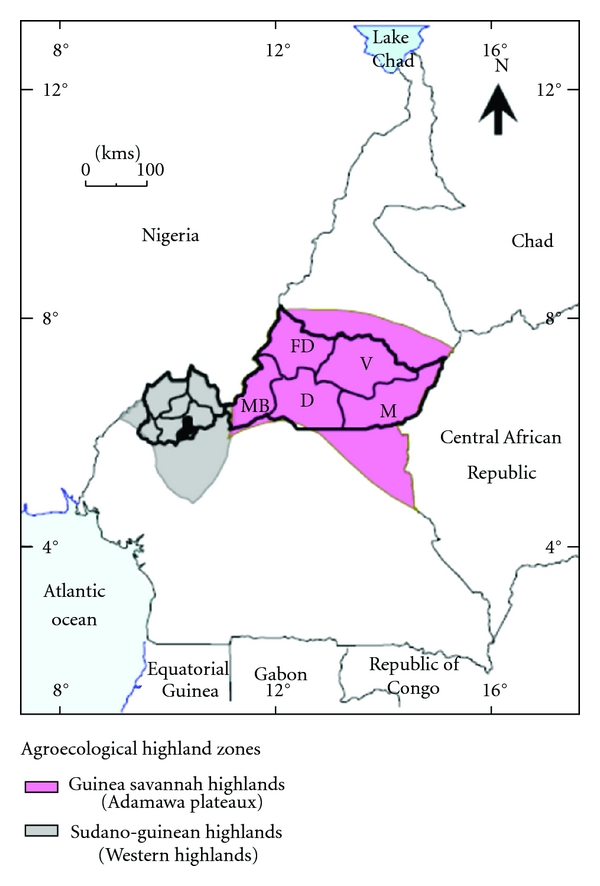
Map of Cameroon showing administrative regions within the Northwest and Adamawa Regions. Divisions in Northwest region are Donga and Matung, Menchum, Bui, Mezam, Boyo, and Ngo-Ketunja (shaded and not used in this study). Divisions in Adamawa region are V: Vina (study area); M: Mbere; D: Djerem; MB: Mayo-Banyo; FD: Faro et Deo.

Cattle populations in the Western highlands (5°–7°N and 10°-11°E) and Adamawa plateaux (6-7°30′N and 12°30′–14°E) of Cameroon (Figure 1) were sampled in the years 2009 and 2010 as part of a bovine TB prevalence study. A SIT bovine TB prevalence rate of 26% recorded by Muchaal [25] in the Western highlands of Cameroon was used to estimate the number of cattle required to detect ≥1 positive reactor with a desired 95% confidence and precision of 5% as previously described [28]. The selection of cattle herds was done by the random-number generation method of cattle keeping communities, cattle owners, and locations of herds from records of annual livestock vaccination campaigns (contagious bovine pleuropneumonia, pasteurellosis, black quarter) at the Regional Delegations of MINEPIA (Ministère de l'Elevage, des Pêches et des Industries Animales (Ministry of Livestock, Fisheries and Animal Industries)). All animals within selected herds were tested except recently calved cows (within 2 months postpartum) and calves less than 6 months old because of immunosuppression in lactating cows and high maternal antibodies in calves that desensitizes them to tuberculin [29, 30].

During March to September 2009, a total of 2,853 cattle (84 herds) were tested in five administrative divisions in the Northwest regions of the Western highlands (Donga and Matung, Menchum, Bui, Mezam and Boyo) and one division in the Adamawa plateaux (Vina) of Cameroon (Figure 1). Similarly, 1,381 cattle (40 herds) were tested during May to September 2010 in Mezam and Bui divisions in the Western highlands which showed high bovine TB prevalence rates in the previous survey and also in the Vina division in the Adamawa plateaux. However, 30–60 minutes prior to the TST carried out in the year 2010, blood was collected from 807 cattle in 20 randomly selected herds of the 40 already chosen herds (1,381 cattle) to extract serum for lateral flow assay of antibovine TB antibodies (Antibovine TB Ab).

Risk assessments were done to avoid hazards to all persons and animals involved in the project. The project approval and ethical clearances were obtained from the required authorities in Cameroon including the National Ethics Committee, regional delegations of MINEPIA in the Northwest and Adamawa regions. The purpose of the study was explained to the targeted participants usually with the assistance of resident veterinarians, local community leaders, and trusted intermediaries. A herd was tested after an informed consent was given by the owner. Apart from minor jugular vein puncture for blood collection, intradermal injections of AT and BT, and procedural restraining manipulations for safety purposes, the animals were not subjected to suffering.

### 2.2. Antibovine Tuberculosis Antibody Assay

About 5 mL of blood was collected by jugular venipuncture of 807 cattle (20 herds) to extract serum for the detection of antibovine TB Ab against the *M. bovis *MPB70 antigen using the rapid lateral-flow test (Anigen Bovine Tb Ab, BioNote Inc., Republic of Korea), as described by the manufacturer. The immunochromatographic assay using recombinant MPB70 antigen as capture and detector in a direct sandwich method detected antibodies (IgM, IgG) against *M bovis*. Briefly, in the ready-to-use disposable test kit, 10 *μ*L of test serum was poured into the sample well, and after 1 minute, 3 drops of developing buffer (provided as part of the kit) were placed in the buffer well. The result was interpreted after 20 minutes. The presence of two purple coloured bands within the result window, the test area and control line, indicated antibodies positive result whereas no band in the test area in addition to a visible control purple line was negative. An invalid test was one where no coloured band was visible within the result window. The appearance of a control colour band, for positive or negative assays, indicated that the test was working properly.

### 2.3. Tuberculin Skin Tests and Classification of Reactors

**Figure 2:**
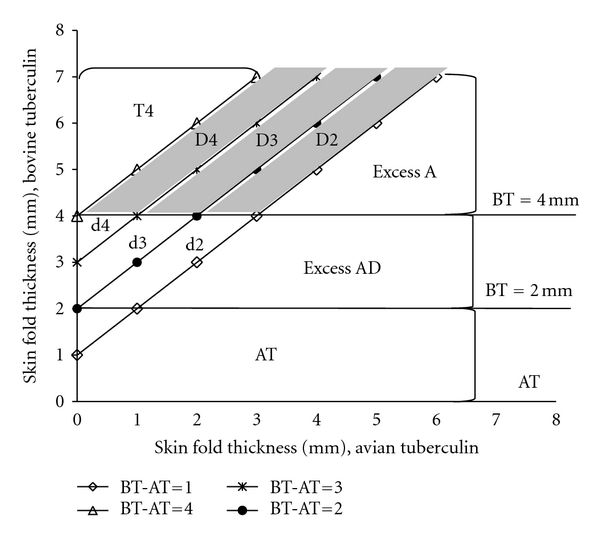
Classification of cattle according to their possible tuberculin skin tests response at ≥4 mm, ≥3 mm, and ≥2 mm cutoff points.

TSTs were carried out in the selected cattle (2,583 in the year 2009 and 1,381 in the year 2010 including the 807 blood donors but after blood collection) by intradermal injections of 0.1 mL each of AT (2500 IU/dose) and BT (3000 IU/dose) in two sites, at 12 cm apart in the right neck region. A correct intradermal injection was confirmed by palpating a small grain-like swelling at each injection site. The skin thickness was measured prior to and 72 hours after injecting the tuberculins using a digital calliper. The OIE-recommended ≥4 mm cutoff point of increase in skin fold thickness [3] as well as ≥3 mm and ≥2 mm cutoff points was assessed for SICCT-BT reactor status. The corresponding ranges ≥1 mm to <4 mm, ≥1 mm to <3 mm, and ≥1 mm to <2 mm were classified as doubtful responses, respectively. SICCT-BT was noted as negative if the skin response was <1 mm. SIT-BT interpretations were done using skin fold thickness of ≥4 mm, ≥2 mm to <4 mm, and <2 mm for positive, doubtful, and negative responses, respectively [3]. These cutoff points were assessed against the demonstrated circulating antibovine Tb antibodies status and classified as adapted from Martrenchar et al. [23] to determine the cutoff zone and risk group of TST reactors for consideration (Figure 2).

### 2.4. Data Management and Statistical Analysis

The lateral flow assay results and TST data at the ≥2 mm, ≥3 mm, and ≥4 mm cutoff points for individual cattle were entered into Microsoft Excel (Microsoft Corporation, USA) and also exported to SigmaPlot (Systat Software Inc, USA) for further analysis. The seroprevalence estimates, rates of TST reactors in the tested cattle population, and agreement between both methods at the predefined cutoff points were assessed [28].

The predictive values and diagnostic likelihood ratios of TST at the various cutoff points were compared against the antibovine TB Ab assay [28]. With sensitivity and specificity values obtained by Ameni et al. [9] and Pollock et al. [12], the observed prevalence rates were corrected using the Rogan-and-Gladen formula [28, 31]. The *kappa *statistics was used to estimate the degree of agreements between both tests while Chi-square techniques were applied to compare individual and herd prevalence of reactors in the different variables [28, 32].

The figure was adapted from Martrenchar et al. [23] where

- BT = (BT_72_–BT_0_) is the skin fold thickness at the injection site of bovine tuberculin at 72 hours;
- AT = (AT_72_–AT_0_) is the skin fold thickness at the injection site of avian tuberculin at 72 hours;
- (D + d) is the SICCT-BT doubtful responses; the skin responses (D2 + d2), (D3 + D2 + d3 + d2), and (D4 + D3 + D2 + d4 + d3 + d2) are for ≥1 mm to <2 mm, ≥1 mm to <3 mm, and ≥1 mm to <4 mm cutoff ranges, respectively;
- Excess d4 (Xd4) = d4 + d3 + d2 is the SICCT-BT doubtful responses (≥4 mm cutoff point) and classified as SIT-BT doubtful responses (when 1 mm ≤ (BT − AT) <4 mm and 2 mm ≤ BT < 4 mm);
- Excess d3 (Xd3) = d3 + d2 is the SICCT-BT doubtful responses (≥3 mm cutoff point) and classified as SIT-BT doubtful responses (when 1 mm ≤ (BT – AT) < 3 mm and 2 mm ≤ BT < 4 mm);
- Excess d2 (Xd2) = d2 is the SICCT-BT doubtful responses (≥2 mm cutoff point) and classified as SIT-BT doubtful responses (when 1 mm ≤ (BT – AT) < 2 mm and 2 mm ≤ BT < 4 mm);
- Excess D4 (XD4) = (D4 + D3 + D2) is the SICCT-BT doubtful responses at ≥4 mm cutoff point and classed as SIT-BT-positive animals (when 1 mm ≤ (BT – AT) < 4 mm and BT ≥ 4 mm);
- Excess D3 (XD3) = (D3 + D2) is the SICCT-BT doubtful responses at ≥3 mm cutoff point and classed as SIT-BT-positive animals (when 1 mm ≤ (BT – AT) < 3 mm and BT ≥ 4 mm);
- Excess D2 (XD2) = (D2) is the SICCT-BT doubtful responses at the ≥2 mm cutoff point and classed as SIT-BT-positive animals (when 1 mm ≤ (BT – AT) < 2 mm and BT ≥ 4 mm);
- T4 is the SICCT-BT-positive animals at ≥4 mm cutoff point (when (BT − AT) ≥ 4 mm);
- T3 = (T4 + XD4 + Xd4) is the SICCT-BT-positive animals at ≥3 mm cutoff point (when (BT − AT) ≥ 3 mm);
- T2 = (T3 + XD3 + Xd3) is the SICCT-BT-positive animals at ≥2 mm cutoff point (when (BT − AT) ≥ 2 mm);
- Excess A (XA) is the animals classed as SIT-BT-positive animals and infected with atypical mycobacteria according to SICCT-AT (when BT ≥ 4 mm and (BT – AT) < 1 mm);
- Excess AD (XAD) is the animals classed as SIT-BT doubtful responses and infected with atypical mycobacteria according to SICCT-AT (when 2 mm ≤ BT< 4 mm and (BT − AT) < 1 mm);
- AT is the animals infected with atypical mycobacteria according to SICCT-AT and classed as SIT-BT negative animals (when BT < 2 mm and (AT − BT) > 0 mm).

## 3. Results

### 3.1. Observed Prevalence Rates and Agreements between Lateral Flow Assay and Tuberculin Skin Tests at ≥2 mm, ≥3 mm, and ≥4 mm Cutoff Points

The observed TST results at modified cutoff points and antibovine TB Ab assay in 807 cattle are summarized in Table 1. Of 807 tested cattle, antibovine TB Ab was detected in 37.17% (95% CI: 30.64–43.71) while 11.77% (95% CI: 9.55–14.00), 8.92% (95% CI: 6.96–10.88), and 3.59% (95% CI: 2.31–4.88) of them were SICCT-BT positive at ≥2 mm, ≥3 mm, and ≥4 mm cutoff points, respectively. The proportion of SICCT-BT/antibovine TB Ab reactors was highest (*P* < 0.05) at the ≥2 mm (9.42% (95% CI: 7.40%–11.43%)) followed by the ≥3 mm (7.93% (95% CI: 6.07–9.79)) and ≥4 mm (3.59% (95% CI: 2.31%–4.88%)) cutoff point groups.

However, analysis of all antibovine TB Ab reactors (300) revealed that 25.33%, 21.33%, 9.67%, and 27% of them were positive at the SICCT-BT ≥ 2 mm, ≥3 mm, and ≥4 mm cutoff points and SIT-BT, respectively. The proportion of SICCT-BT doubtful/antibovine TB Ab positive reacting cattle was highest (*P* < 0.05) at the SICCT-BT ≥ 4 mm (21%) followed by the ≥3 mm (5.67%) and ≥2 mm (1.67%) cutoff point groups. However, 0.62% (95% CI: 0.08%–1.16%), 3.47% (95% CI: 2.21%–4.73%), and 8.80% (95% CI: 6.84%–10.75%) of the 807 tested cattle showed SICCT-BT inconclusive results while 0.62% (95% CI: 0.08%–1.16%), 2.11% (95% CI: 1.12%–3.10%), and 7.81% (95% CI: 5.96%–9.66%) reactors were SICCT-BT doubtful and antibovine TB Ab positive at the o 2 mm, ≥3 mm, and ≥4 mm cutoff points, respectively. Over 27.14% (95% CI: 24.07%–30.21%) negative SICCT-BT reactors were also positive for antibovine TB Ab.

Furthermore, 13.14% (95% CI: 10.80%–15.47%) SIT-BT and 10.04% (95% CI: 7.96–12.11) SIT-BT positive/antibovine TB-Ab-positive animals were recorded. Among the SIT-BT reactors, 76.42% of them were antibovine TB Ab reactors and over 89.62%, 67.92%, and 27.36% were SICCT-BT reactors while 71.70%, 60.38%, and 27.36% were SICCT-BT-positive/antibovine TB-Ab-positive animals at the 2 mm; ≥3 mm, and ≥4 mm cutoff points, respectively. Overall, 31 (3.84%) SICCT-BT doubtful/SIT-BT-positive animals at superior cutoff points were classified as SICCT-BT reactors at ≥3 mm (2.97%) and ≥2 mm (3.84%) cutoff points (Table 1).

The agreement between TST at modified cutoff points and antibovine TB antibody assay was shown in Table 2. In all, the concordances (TST positive/antibovine TB Ab positive) were 100%, 88.89%, 80%, and 76.42% in positive subjects at SICCT-BT ≥4 mm, ≥3 mm, and ≥2 mm cut-offs and SIT-BT, respectively. The discordances (TST negative/antibovine TB Ab positive) were 34.83%, 32.11%, 31.46%, and 31.24%, at the SICCT-BT ≥4 mm, ≥3 mm, and ≥2 mm cutoff points and SIT-BT, respectively. However, the concordances (TST positive/antibovine TB Ab positive) in antibovine TB Ab positive subjects were 9.67%, 21.33%, 25.33%, and 27% while the discordances (TST negative/antibovine TB Ab positive) were 94%, 78.67%, 74.67%, and 73%, at the SICCT-BT ≥4 mm, ≥3 mm, and ≥2 mm cutoff points and SIT-BT, respectively. The bench marks (>0.80: very good agreement; 0.61–0.80: good agreement; 0.41–0.60: moderate agreement; 0.21–0.40 fair agreement and ≤0.20: poor agreement) for evaluating points estimates of* kappa *values [28] revealed a poor agreement between SICCT-BT test and antibovine TB Ab assay at the ≥4 mm skin response cutoff point and fair agreements at the other cutoff points (≥3 mm and ≥2 mm; and SIT-BT).

### 3.2. Comparison of Tuberculin Skin Tests at Modified Cutoff Points and Lateral Flow Assay in Cattle Reactors

The predictive values and likelihood ratios of SICCT-BT at various cut-off values and SIT-BT in cattle reactors against the antibovine TB Ab assay are shown in Table 3. Strong associations were noted between the seroprevalence estimates and rates of TST reactors irrespective of the TSTS cut-off value (*P* < 0.05; *χ* ^2^ > 48) in this study. However, decreasing the cutoff points revealed inverse relationships with test predictive values and diagnostic likelihood ratios. The ability of SICCT-BT to produce no false negative result increased with increase in cutoff point (nonsignificant differences were noted between the ≥2 mm versus ≥3 mm and ≥3 mm versus ≥4 mm cutoff points). The findings also suggested that prediction of disease status improved with severe interpretation of TST (decreasing cutoff point). The study indicated that using antibovine TB Ab assays as ancillary diagnostic tests to SICCT-BT in cattle could significantly improve diagnosis of bovine TB cases. Statistically, the best all round SICCT-BT performance was realized at the ≥3 mm cutoff point. However, the ≥2 mm cut-off value showed the highest positive predictive value and a comparable positive diagnostic likelihood ratio to the others.

**Figure 3:**
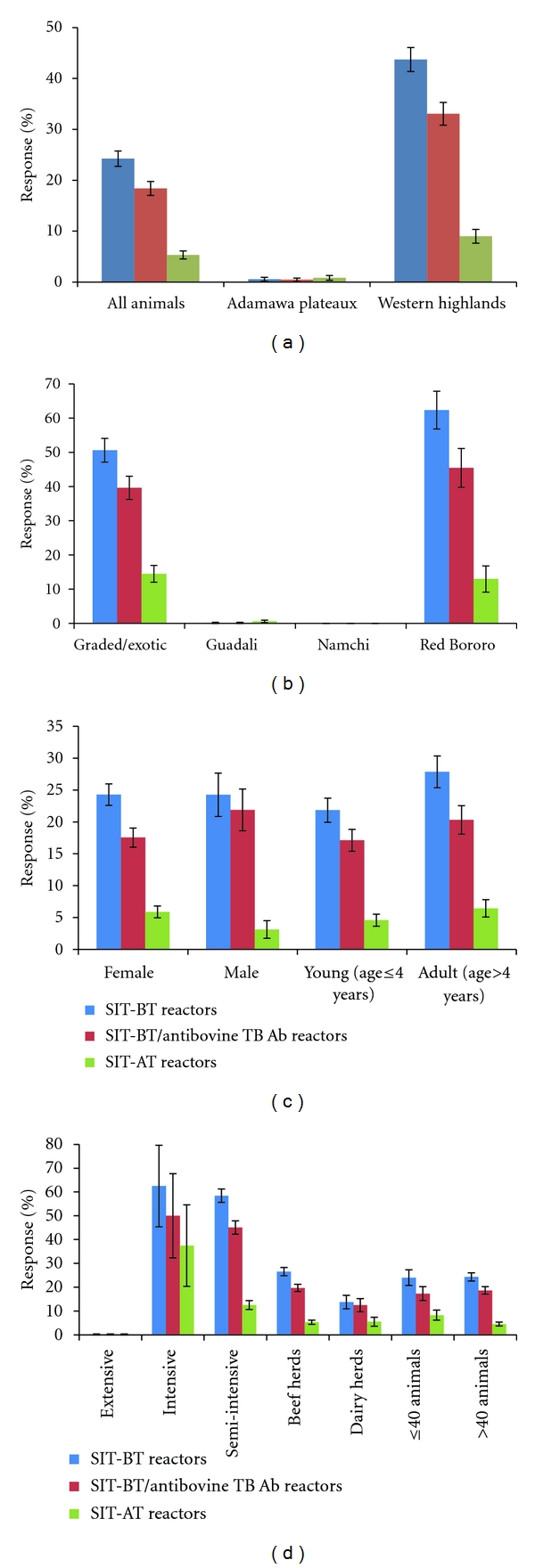
Detection of antibovine TB antibody and SIT-BT reactors in 807 tested cattle according to (a) study location, (b) breed, (c) sex and age group, and (d) management systems and herd sizes.

**Figure 4:**
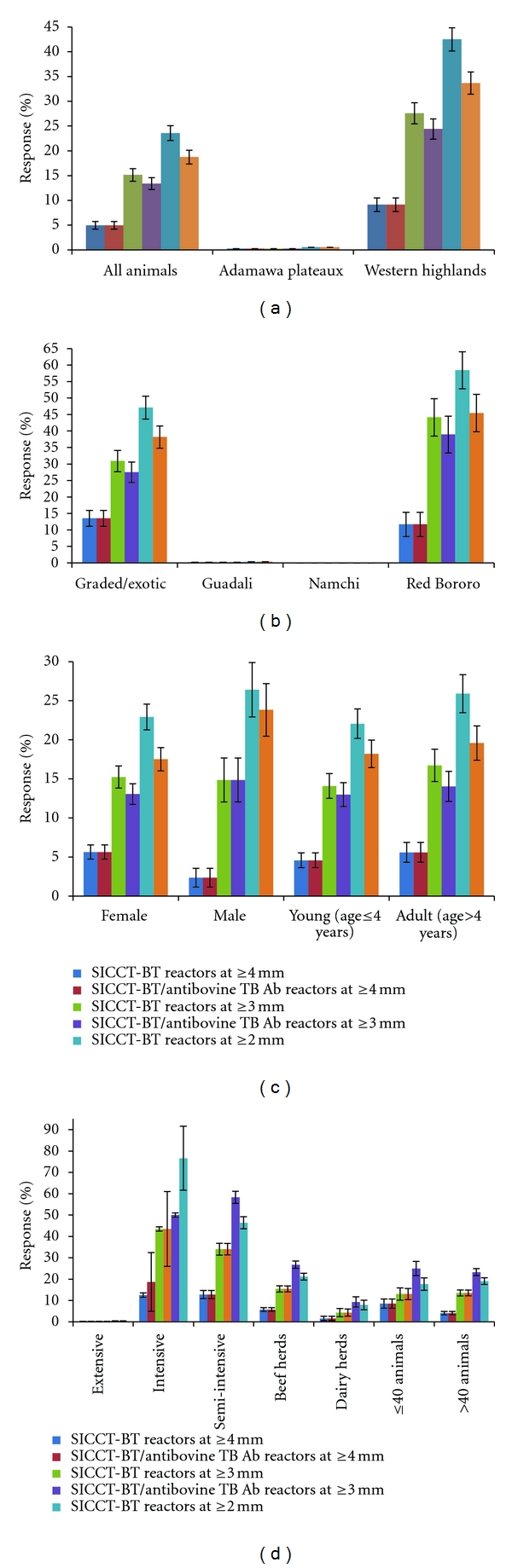
Detection of antibovine TB antibody and SICCT-BT reactors in 807 tested cattle at the ≥4 mm, ≥3 mm, and ≥2 mm cutoff points according to (a) study location, (b) breed, (c) sex and age group, and (d) management systems and herd sizes.

The detection of antibovine TB Ab positive cattle and proportions of SICCT-BT reactors and antibovine TB Ab/SICCT-BT reactors at the different cut-offs are shown in Figure 4. The SICCT-BT ≥ 2 mm cutoff value gave the highest (*P* < 0.05) rate (23.60%) followed by the ≥3 mm (15.15%) and ≥4 mm (4.98%) cutoff points. Overall, similar trends were observed for SICCT-BT and antibovine TB-Ab-positive/SICCT-BT-positive animals for the parameters considered. In all, 16.78% SIT-BT- and 12.73% SIT-BT-positive/antibovine TB-Ab-positive animals were detected (Figure 3). Among the SIT-BT reactors, over 98.59%, 61.23%, and 10.38% were SICCT-BT reactors and 78.88%, 60.19%, and 10.38% were SICCT-BT-positive/antibovine TB-Ab-positive animals at the ≥2 mm, ≥3 mm, and ≥4 mm cutoff points, respectively. Also, 84.07% SICCT-BT-positive/antibovine TB-Ab-positive animals were identified among the SIT-BT reactors, irrespective of the interpreting SICCT-BT cutoff point. SIT-AT positive reacting cattle was widespread in the study.

Furthermore, antibovine TB Ab assay revealed that over 95% (95% CI: 75.1%–99.9%) of the test herds had ≥1 antibovine TB-Ab-positive animal, while SIT-BT and SICCT-BT at ≥2 mm cutoff point gave nonsignificantly higher TST positive/antibovine TB Ab positive herds (36.84%, (95% CI: 16.3%–61.6%)) than SICCT-BT at ≥3 mm and ≥4 mm (30%, (95% CI: 12.6%–56.5%)) cutoff points. Indeed, the herd infection (i.e., ≥1 TST positive animal) rates were 35% (95% CI: 15.4%–59.2%) for SIT-BT and SICCT-BT ≥2 mm cutoff point and 30% (95% CI: 11.9%–54.3%) for the SICCT-BT at ≥3 mm and ≥4 mm cutoff points. Similarly, higher but comparable herd infection rates were obtained when severe interpretations were considered for complete TST screening of 1,381 cattle in 40 herds (i.e., for SICCT-BT: 40% (95% CI: 24.9%–56.7%) at ≥3 mm and ≥4 mm cut-offs; 45% (95% CI: 29.3%–61.5%) at ≥2 mm cut-off and also 47.5% (95% CI: 33.8%–66.2%) for SIT-BT). Also, significantly higher (*P* < 0.05) SICCT-BT- and SIT-BT-infected herds were recorded in the Western highlands (48.39% (95% CI: 30.2%–66.9%) at the SICCT-BT ≥4 mm and ≥3 mm cutoff points; 51.61% (95% CI: 33.1%–69.8%) at the SICCT-BT ≥2 mm cutoff point and 54.84% (95% CI: 36%–72.7%) for SIT-BT) than in the Adamawa plateaux (11.11% (95% CI: 24.9%–56.7%) for the SICCT-BT ≥4 mm and ≥3 mm cutoff groups and 22.22% (95% CI: 2.8%–60%) for the SICCT-BT ≥2 mm cut-off and SIT-BT groups). 

### 3.3. Prevalence Rates of Bovine Tuberculosis in Previously Tested Cattle at the Modified Cutoff Points

The TST survey in the year 2009 (2,853 cattle) and complete data of 2010 (1,381 cattle) were reanalysed using the predefined cutoff points (Tables 4 and 5). Overall, the prevalence rates and trends of bovine TB in both surveys were very similar. The differences in the prevalence of SICCT-BT reactors were significantly higher between the cutoff points (≥4 mm versus ≥3 mm: *χ* ^2^ = 46.021; *P* ≤ 0.001; ≥4 mm versus ≥2 mm: *χ* ^2^ = 64.015; *P* ≤ 0.001; ≥3 mm versus ≥2 mm: *χ* ^2^ = 16.056; *P* ≤ 0.001). Age, sex, breed, animal site, and husbandry systems were significant (*P* < 0.05) risk factors to the epidemiological status of bovine TB in the regions. 

## 4. Discussion

There is gross inadequacy in the implementation of the existing bovine TB control policy in Cameroon. Culling of TST reactors as part of a national animal disease control policy is not a routine practice due to political, economic, and social limitations. However, veterinarians continue to identify bovine TB lesions in slaughtered cattle across the country [33–35]. TB lesions have been detected in TST reactors at cutoff points less than the OIE-recommended optimal 4 mm cut-off [8, 9, 15] and TST negative reactors [10]. TB lesions were also observed in TST doubtful and negative reactors in Mezam Division in the present study. Lack of knowledge on the actual magnitude and distribution of the disease, inadequate laboratories and field expertise, and politicoeconomic deficiencies are common factors that limit bovine TB control in most of Africa [36]. The current control approach in Cameroon is based on controlling animal movements, culling suspected bovine TB cases and carcass condemnation (partial or whole) at meat inspection [37]. Apparently, the strategies were designed to reduce the general prevalence and monitor spread of the disease in livestock. TST is presently a passive component of Cameroon's government strategy to control bovine TB which is of major concern to the veterinary and medical services.

Maximum detection of bovine TB in cattle populations in Cameroon is vital to understand its epidemiology and zoonotic potentials and also achieve significant reduction and control of the disease in livestock. Cell-mediated immune responses develop early after bovine TB infection in cattle while antibody responses may not become obvious until later and at advanced stages of the disease, when cell mediated reactions (TST reactions) are waning [38–40]. TST can boost antibody responses in *M. bovis *infected cattle and emphasizes the importance of timing of collection of blood samples on the interpretation the test [38]. In this study, the antibovine TB antibody detection (Anigen lateral-flow assay) that employed recombinant *M. bovis *MPB70 antigen as capture and detector was conducted prior to TST. This antibovine TB antibody test kit has a sensitivity of 90% against bovine TB confirmed by bacterial isolation and a sensitivity of 85.1% and specificity of 98.6% against TST [41]. Also using the Anigen lateral-flow assay, Whelan et al. [42] achieved a sensitivity of 84% and a specificity of 84.2% for serological diagnosis of *M. bovis *infection in cattle. Similar and relatively high sensitivity (86.5% and 84.6%) and specificity (83.8% and 91.4%) have been reported with other lateral flow techniques (CervidTB STAT-PAK and DPP VetTB assays, resp.) for the rapid diagnosis of bovine TB in farmed Red deers [43]. Furthermore, a sensitivity of 89.6% and specificity of 90.4% were achieved in the diagnosis of *M. bovis *infection in Eurasian wild boar using the DPP VetTB assay (based on combining two separate test antigens) [44]. However, the specificity of these test kits could be affected by cross-reacting members of the *M. avium *complex [43, 44], and high false positive results were observed when a commercial multiantigen lateral flow assay was performed in dairy cattle [45]. Nonetheless, significantly higher specificity of 98.4% and sensitivity of 93.1% in the diagnosis of bovine TB in cattle have been obtained for multiplex immunoassay based on a combination of antigens compared to those of assays based on a single antigen [22, 42]. The TST accuracy against postmortem detection of TB lesions revealed a sensitivity of 86% and specificity of 90% for SIT-BT [12], while sensitivity values of 69%, 65%, and 59% at SICCT-BT ≥2 mm, ≥3 mm, and ≥4 mm cutoff points and a specificity of 97% at these cutoff points have been reported [9]. The lack of a well-established gold standard in this study was a key problem in calculating the sensitivity and specificity of the lateral flow assay and TST at the modified cutoff points.

The findings of this study suggest that TST at any cutoff point could be used to detect bovine TB in cattle and the test accuracy increased with increase in cut-off value. Cattle presenting differential SICCT-BT skin thickness of less than 4 mm in Cameroon should therefore not be excluded that they are negative for bovine TB. These animals may be infected but low reacting or not reacting at all if their immune systems were not stimulated enough for a positive response at the ≥4 mm cutoff point [46, 47] due to conditions such as stress that compromise immune function [48]. Also, the animals may have been sensitized to environmental mycobacteria [38]. Furthermore, delayed hypersensitivity to tuberculin may not develop for a period of 3–6 weeks following infection [3, 10]. Delaying TST of a herd/animal suspected to have been in contact very recently with infected animals in order to reduce the probability of false-negatives has been suggested [10] since it is unlikely that the control and eradication of TB from a herd will be achieved with only a single tuberculin test [3]. In this study, maximum positive prediction values and negative likelihood ratio were observed at the SICCT-BT ≥2 mm cutoff point and maximum negative prediction and positive likelihood ratio at the ≥4 mm cutoff point. The findings also revealed that 31 cattle (over 3.84%) considered as SICCT-BT doubtful reactors at the ≥4 mm cutoff point could be identified as positive bovine TB cases at the ≥3 mm and ≥2 mm cutoff points. The poor to fair agreements recorded suggested that severe interpretation of TST (i.e., decreasing skin response cut-off values) improved the agreement between TST and the lateral flow assay to detect TST positive reactors. The prevalence rates at the modified cutoff points could have influenced the estimated *Kappa* values. However, low *kappa* values have been obtained between good diagnostic and negatively correlated tests [28]. The poor correlation between comparative TST at the ≥4 mm cutoff point and antibovine TB antibody test results in the study was not unexpected. Therefore, the importance of determining appropriate localised TST cut-off values supported by validated methods in Cameroon cannot be overemphasized.

Though it is essential that tuberculin of sufficient potency to produce a reaction in the maximum number of infected animals is essential, a tuberculin of potency greater than that to which the majority of infected animals will respond has been proposed in TST [10]. However, Good and Duignan [10] had warned that highly potent tuberculin tends to increase the frequency of reactions associated with cross-sensitisations arising from other organisms such as the human and avian types (*M. tuberculosis *and *M. avium*, resp.) and other (nonpathogenic) mycobacteria. Nonspecific responses in TST due to atypical or environmental mycobacteria have been widely reported [2, 3, 49–51]. Indeed, Lesslie et al. [52–54] recorded hypersensitivity responses to avian tuberculin that was equal or higher than responses to bovine tuberculin in cattle naturally infected with *M. bovis* and presenting visible lesions at slaughter. Therefore, severe interpretations of TST reactions should be employed when EU- and OIE-recommended tuberculin preparations are used in bovine TB endemic regions and environments where multiple mycobacteria are coexisting. The findings of this study agree with Martrenchar et al. [23] who reported high frequency of atypical mycobacteria which severely limited the reliability of SIT-BT and SICCT-BT results at the OIE-recommended 4 mm cutoff point in Northern Cameroon. Severe interpretations of TST results in the study revealed that many SIT-BT positive and SICCT-BT doubtful responses at ≥4 mm cutoff point could be appropriately identified as bovine TB cases at reduced cutoff points (some Excess D4 and Excess D3 reactors). The high detection of TST and antibovine TB antibody positive herds irrespective of TST cutoff point and findings of circulating antibovine TB antibody could suggest that the cattle were widely exposed to and affected bovine TB and other mycobacterial infections.

In this study, reducing the cutoff point from ≥4 mm improved the *ante mortem* detection of bovine TB in cattle using SICCT-BT and antibovine TB Ab tests. Overall, the maximum test ability was realized at ≥3 mm cutoff point and the best SICCT-BT positive predictive value was at ≥2 mm cutoff point. These findings revealed that interpreting SICCT-BT at the ≥2 mm cutoff point, and not at the ≥3 mm or ≥4 mm cutoff points, was beneficial from a public health perspective. However, there would be concrete risk of unnecessarily identifying more cattle at severe TST interpretations. This study cannot exclude that some SICCT-BT doubtful reactors at the ≥3 mm and the ≥4 mm cutoff points were infected cases detected at the ≥2 mm cutoff point. The application of the SICCT-BT ≥2 mm cutoff point should be considered in cattle in the agro-ecological highland environments of Cameroon for greater detection of bovine TB. Severe TST interpretation would be vital to effective control of the disease and reduction of its zoonotic risks to public health and food safety in the country.

## 5. Conclusion

 The TST and antibovine TB antibody tests when used in parallel offered improved detection of bovine TB compared to individual tests. Bovine TB was detected at all the cutoff points and there were strong associations between both methods in the highlands of Cameroon. The best test performance was realized at the ≥3 mm cutoff point. However, interpreting SICCT-BT at ≥2 mm cutoff point was more strategic from a public health context since more affected cases would be predicted. The study revealed that the prevalence of bovine TB was high and atypical mycobacteria infection was widespread in the regions. Bovine TB-infected cattle which maybe anergic due to age, malnutrition, and/or suffering from concurrent diseases such as internal and external parasitosis (common scenarios in the study regions) could be detected at severe SICCT-BT interpretation. Their delayed hypersensitivity responses to tuberculin would be limited and cannot express the full OIE-recommended ≥4 mm cutoff point. However, it is important to investigate the performance of TST at modified cutoff points against defined bovine TB status confirmed by postmortem examination and culture of TB lesions in reacting animals in the Cameroon environments.

## Figures and Tables

**Table 1:** Distribution of reactors of tuberculin skin tests at various cutoff points and antibovine tuberculosis antibody assay (according to region, sex, age, and herd size) in cattle in Cameroon.

Variable/Label	Number of animals tested	SICCT-BT reactors (%)			SIT-BT reactors	SICCT BT doubtful/SIT-BT positive reactors (%)			SICCT BT doubtful/SIT BT doubtful responses (%)			Excess A* (%)	% of SICCT-BT doubtful and classed positive at inferior cutoff points (%)		Antibovine TB Ab reactors^#^ (%±SE)
		T4	T3	T2		XD4	XD3	XD2	Xd4	Xd3	Xd2		XD4 at ≥3 mm	XD4 + XD3 at ≥2 mm	
All animals	807	3.59	8.92	11.77	13.14	3.72	0.87	0.50	5.08	2.60	0.12	4.46	2.97	3.84	37.17 ± 3.33
*Agroecological Regions*															
Adamawa plateaux	363	0.28	0.28	0.55	0.55	0.28	0.28	0.00	0.28	0.28	0.28	0.00	0.00	0.28	29.75 ± 4.70
Western Highlands	444	6.31	15.99	20.95	23.42	6.53	1.35	0.90	9.01	4.50	0.00	8.11	5.41	6.76	43.24 ± 4.61
*Sex and Age*															
Female	647	4.02	8.96	11.44	13.14	4.17	1.08	0.62	4.64	2.16	0.15	4.48	3.25	4.33	36.32 ± 3.71
Male	160	1.88	8.75	13.13	13.13	1.88	0.00	0.00	6.88	4.38	0.00	4.38	1.88	1.88	40.63 ± 7.61
Young (o 4 years)	481	3.33	8.32	11.02	11.85	3.33	0.42	0.21	4.99	2.49	0.00	3.53	2.91	3.33	38.46 ± 4.35
Adult (>4 years)	326	3.99	9.82	12.88	15.03	4.29	1.53	0.92	5.21	2.76	0.31	5.83	3.07	4.60	35.28 ± 5.19
*Herd sizes*															
Animals ≤ 40	169	5.92	10.06	12.43	13.02	6.51	1.18	0.59	2.96	1.78	0.00	4.14	5.92	7.10	28.99 ± 6.84
Animals > 40	638	2.98	8.62	11.60	13.17	2.98	0.78	0.47	5.64	2.82	0.16	4.55	2.19	2.98	39.34 ± 3.79

T4, T3, T2, XD4, XD3, XD2, Xd4, Xd3, Xd2, and Excess A are as defined in Figure 2.

SICCT-BT: Single Intradermal Comparative Cervical Tuberculin skin test for the detection of bovine tuberculosis.

SIT-BT: Single Intradermal Tuberculin skin test for the detection of bovine tuberculosis.

Antibovine TB Ab: Antibovine tuberculosis antibody assay.

^#^ *Breed*: Upgraded/Exotic = 42.03 ± 6.72; Gudali = 31.30 ± 4.10; Namchi = 22.58 ± 14.72; Red Bororo = 67.53 ± 10.46.

*Management and production system*: Extensive = 31.76 ± 4.13; Intensive = 75.00 ± 30.01; Semi-intensive = 44.69 ± 5.53;

*Beef herds* = 38.07 ± 3.70; Dairy herds = 33.10 ± 7.66.

**Table 2:** Agreement between reactors of tuberculin skin tests and antibovine tuberculosis antibody assay according to various tuberculin skin response cutoff points.

	SICCT-BT cutoff points						SIT-BT	
	≥4 mm		≥3 mm		≥2 mm		≥4 mm	
	Number	%	Number	%	Number	%	Number	%
TST positive/Anti-BTB Ab positive	29	3.59	64	7.93	76	9.42	81	10.04
TST negative^#^/Anti-BTB Ab positive	271	34.82	236	29.24	224	27.76	219	27.14
TST positive/Anti-BTB Ab negative	0	0	8	0.99	19	2.35	25	3.10
TST negative^#^/Anti-BTB Ab negative	507	62.83	499	61.83	488	60.47	482	59.73

Total	807		807		807		807	

Agreement	29/807	3.59	64/807	7.93	76/807	9.42	81/807	10.04

*Kappa* statistics*	0.119		0.234		0.251		0.254	

TST: Tuberculin skin test.

Anti-BTB Ab: antibovine tuberculosis antibody assay.

^#^Not TST positive including TST doubtful reactors.

*Kappa ranges from 1 (complete agreement beyond chance) to 0 (agreement is equal to that expected by chance), whereas negative values indicate that agreement less than that is expected by chance.

**Table 3:** Predictive values and likelihood ratios at the ≥2 mm, ≥3 mm, and ≥4 mm cutoff points for tuberculin skin tests and lateral flow assay of cattle reactors in Cameroon.

Cutoff point	Test predictive value; % (95% CI)		Diagnostic likelihood ratio; (95% CI)	
	Positive result	Negative result	LR+	LR−
(a) For SICCT-BT test against antibovine TB Ab assay				

≥2 mm	34.05 (29.16–38.50)	94.41 (91.66–96.41)	2.54 (2.03–3.08)	0.29 (0.45–0.18)
≥3 mm	29.55 (25.32–33.13)	97.58 (95.42–98.79)	2.77 (2.24–3.27)	0.16 (0.32–0.08)
≥4 mm	14.67 (12.15–15.94)	100 (98.88–100)	2.87 (2.31–3.17)	0* (0.19–0)

(b) For SIT-BT test against antibovine TB Ab assay				

≥4 mm	33.03 (28.13–37.61)	93.53 (90.87–95.58)	2.45 (1.94–2.99)	0.34 (0.50–0.23)

*The perfect diagnostic test would be expected to have an LR− equal to zero and an LR+ equal to infinity (producing no false negatives, but detecting all negatives and detecting all positives, and generating no false positives). The best test therefore for excluding a disease is the one with the lowest LR− and the test with the highest LR+ is the best for detecting disease [28].

**Table 4:** Prevalence of SICCT reactors in 1,381 cattle tested in the year 2010 at modified cutoff points and SIT reactors in the highlands of Cameroon.

Variable	No animals tested	SICCT-BT reactors % (95% CI)			SICCT-AT reactors*; %(SE)	SIT-BT reactors; %(95% CI)	SIT-AT reactors*; %(SE)
		≥4 mm	≥3 mm	≥2 mm	≥4 mm	≥4 mm	≥4 mm
All animals	1,381	7.41 (6.02–8.79)	13.25 (11.47–15.04)	17.26 (15.36–19.25)	0.65 ± 0.42	18.35 (14.35–22.35)	7.46 ± 1.39
*Agroecological location*							
ADP	363	0.43^a^ (0–1.11)	0.40^a^ (0–1.04)	0.79^a^ (0–1.70)	0.55 ± 0.76	0.59^a^ (0–2.13)	0.83 ± 0.93
WHC	1,018	9.89^b^ (8.06–11.72)	17.84^b^ (15.49–20.19)	23.13^b^ (20.54–25.72)	0.69 ± 0.51	24.68^b^ (19.49–29.88)	9.82 ± 1.83
*Breed*							
Upgraded/Exotic	764	10.87^a^ (8.66–13.08)	16.12^a^ (13.52–18.73)	21.07^a^ (18.18–23.96)	0.79 ± 0.63	24.03^a^ (18.09–29.96)	11.39 ± 2.25
Guadal	492	0.31 (0–0.79)	0.28 (0–0.75)	0.57 (0–1.24)	0.41 ± 0.56	0.40 (0–1.50)	0.61 ± 0.69
Namchi	31	0	0	0	0	0	0
Red Bororo	94	18.84^a^ (10.94–26.75)	62.22^b^ (52.42–72.02)	79.29^b^ (71.10–87.49)	1.06 ± 2.07	72.28^b^ (54.54–90.01)	13.83 ± 6.98
White Fulani							
*Sex and Age*							
Female	1,107	8.29^a^ (6.66–9.91)	13.90^a^ (11.87–15.94)	17.69 (15.44–19.94)	0.63 ± 0.47	19.73^a^ (15.14–24.33)	8.49 ± 1.64
Male	274	3.83^b^ (1.56–6.11)	10.62^a^ (6.98–14.28)	15.51 (11.22–19.79)	0.73 ± 1.01	12.77^a^ (5.02–20.51)	3.28 ± 2.11
Age ≤ 4 years	716	4.41^c^ (2.91–5.91)	9.71^b^ (7.55–11.88)	12.50^a^ (10.07–14.92)	0.28 ± 0.39	11.93^b^ (7.28–16.59)	4.05 ± 1.44
Age > 4 years	665	10.63^d^ (8.29–12.97)	17.07^c^ (14.21–19.92)	22.38^b^ (19.22–25.55)	1.05 ± 0.78	25.26^c^ (18.79–31.73)	11.13 ± 2.39
*Management system*							
Extensive	488	0.31 (0–0.80)	0.28 (0–0.75)	0.58 (0–1.25)	0	0.41 (0–1.51)	0.20 ± 0.40
Intensive/Zero grazing	552	8.95^a^ (6.57–11.34)	12.03^a^ (9.31–14.74)	16.50^a^ (13.40–19.59)	1.09 ± 0.87	20.74^a^ (14.11–27.36)	10.87 ± 2.60
Semi-intensive	341	15.05^b^ (11.26–18.85)	33.89^b^ (28.78–38.83)	42.36^b^ (37.11–47.60)	0.88 ± 0.99	40.17^b^ (29.97–50.37)	12.32 ± 3.49
Beef herds	692	7.39^c^ (5.44–9.34)	16.40^c^ (13.64–19.16)	20.63^c^ (17.61–23.64)	0.43 ± 0.49	18.97^c^ (13.25–24.70)	5.49 ± 1.70
Dairy herds	689	15.15^d^ (12.48–17.83)	10.10^d^ (7.85–12.35)	13.87^d^ (11.29–16.45)	0.87 ± 0.69	17.73^c^ (12.14–23.32)	9.43 ± 2.18
*Herd size (No animals per herd)*							
≤40 animals	713	9.41^a^ (7.27–11.55)	12.72^a^ (10.27–15.17)	16.38^a^ (13.67–19.11)	1.12 ± 0.77	19.14^a^ (13.48–24.81)	9.96 ± 2.20
>40 animals	668	5.26^b^ (3.57–6.96)	13.82^a^ (11.21–16.44)	18.18^a^ (15.26–21.11)	0.15 ± 0.29	17.50^a^ (11.86–23.15)	4.79 ± 1.62

^a, b, c, d^Label in a category with the different letters in a column are significantly different (*P* < 0.05).

*Observed prevalence.

ADP: Adamawa plateaux of Cameroon.

WHC: Western highlands of Cameroon.

SICCT-BT: Single Intradermal Comparative Cervical Tuberculin skin test for the diagnosis of bovine TB.

SIT-BT: Single Intradermal Tuberculin skin test for the diagnosis of bovine TB.

**Table 5:** Prevalence of SICCT-BT reactors in 2,853 cattle tested in the year 2009 at modified cutoff points in the highlands of Cameroon.

Variable	Animals tested	SICCT-BT reactors % (95% CI)		
		≥4 mm	≥3 mm	≥2 mm
All animals	2,853	7.48 (6.51–8.44)	11.52 (10.35–12.69)	12.92 (11.69–11.15)
*Agroecological location*				
ADP	727	4.10^b^ (2.66–5.54)	5.32^b^ (3.69–6.95)	7.07^a^ (5.21–8.93)
WHC	2,126	8.63^a^ (6.51–8.44)	13.64^a^ (12.18–15.10)	14.92^b^ (13.40–16.43)
*Breed*				
Upgraded/Exotic	368	12.49^a^ (9.12–15.87)	19.39^a^ (15.35–23.43)	21.05^a^ (16.88–25.21)
Guadali	1,317	6.01^b^ (4.73–7.30)	10.32^b^ (8.68–11.96)	12.32^b^ (10.54–14.09)
Namchi	33	3.03	3.03	3.03
Red Bororo	487	11.62^a^ (8.77–14.46)	15.64^a^ (12.42–18.87)	16.52^a^ (13.22–19.82)
White Fulani	648	4.60^b^ (2.99–6.22)	6.72^b^ (4.80–8.65)	7.23^b^ (5.24–9.23)
*Sex and Age*				
Female	2,212	7.73^a^ (6.62–8.85)	12.30^a^ (10.93–13.67)	13.92^a^ (12.48–15.36)
Male	641	6.60^a^ (4.67–8.52)	8.83^b^ (6.63–11.02)	9.45^b^ (7.19–11.72)
Age ≤ 4 years	1,481	5.82^b^ (4.63–7.01)	8.40^c^ (6.99–9.82)	9.72^c^ (8.21–11.22)
Age > 4 years	1,372	9.27^c^ (7.73–10.80)	14.88^d^ (13.00–16.77)	16.37^d^ (14.41–18.33)
*Management system*				
Extensive	1510	6.77^a^ (5.50–8.03)	9.32^a^ (7.85–10.78)	9.93^a^ (8.42–11.44)
Intensive	138	6.38^a^ (2.03–10.46)	17.62^b^ (11.27–23.98)	19.81^b^ (13.16–26.46)
Semi-intensive	1205	8.49^a^ (6.92–10.07)	13.58^b^ (11.64–15.51)	15.87^b^ (13.81–17.93)
Beef herds	2,357	8.16^b^ (7.05–9.26)	10.78^c^ (9.53–12.03)	11.71^c^ (10.41–13.00)
Dairy herds	496	4.24^c^ (2.47–6.02)	15.03^d^ (11.88–18.17)	18.67^d^ (15.24–22.10)
*Herd size (No animals per herd)*				
≤40 animals	1,325	9.19^a^ (7.64–10.75)	11.98^a^ (10.23–13.72)	13.51^a^ (11.67–15.35)
>40 animals	1,528	5.99^b^ (4.80–7.18)	11.12^a^ (9.55–12.70)	12.40^a^ (10.75–14.06)

^a, b, c, d^Label in a category with different letters in a column are significantly different (*P* < 0.05).

SICCT-BT: Single Intradermal Comparative Cervical Tuberculin skin test for the diagnosis of bovine tuberculosis.
